# Two “faces” of e-sports players: the relationship between facial width-to-height ratio and aggressive behavior in the virtual world

**DOI:** 10.3389/fpsyg.2025.1620182

**Published:** 2025-11-19

**Authors:** Zhengda Xu, Xiaohan Li, Song Lin

**Affiliations:** 1 Business School, Beijing Technology and Business University, Beijing, China; 2 Business School, Central University of Finance and Economics, Beijing, China

**Keywords:** facial features, virtual characters, virtual identity, aggressive behavior, e-sports

## Abstract

**Introduction:**

The growing literature has emphasized the role of facial structures in affecting human behavioral tendencies, particularly the debate on whether a person’s facial width-to-height ratio (fWHR) influences their aggressive behavior. The development of the digital economy and e-sports—where players use virtual characters rather than engaging in physical fighting—provides a new research context. We aimed to explore the role of the fWHR of players, and the similarity to their virtual characters, in predicting aggressive behavior.

**Methods:**

We utilized archival data from 954 professional *League of Legend* (LoL) players covering the period from 2017 to 2022. We analyzed the association between players’ fWHR and in-game indicators of aggressive behavior. We also assessed the effect of facial similarity between the player and their virtual character.

**Results:**

We present evidence that e-sports players’ fWHR is not significantly associated with their aggressive behavior in the virtual world. However, we found that players performed more aggressively when their real face was more similar to their virtual character’s face.

**Discussion:**

Our studies extend the boundary of fWHR and aggressive behavior research in the digital era.

## 1 Introduction

Faces can convey a lot of information. In recent years, many scholars have argued that facial width-to-height ratio (fWHR), as a typical human characteristic, has an impact on various behavioral tendencies of people, such as threat behavior (Geniole et al., 2015), deception and exploitation (Geniole et al., 2014; Haselhuhn and Wong, 2011; Stirrat and Perrett, 2010), trait dominance (Carré and McCormick, 2008), physical aggression (Goetz et al., 2013), and overall psychopathy (Anderl et al., 2016). This effect is more pronounced in male athletes in sports, as some scholars have suggested that fWHR is affected by the levels of testosterone in men (Lefevre et al., 2013). Thus, scholars have conducted lots of research using the samples of traditional sports players, such as professional hockey players (Deaner et al., 2012), MMA or UFC fighters (Třebický et al., 2015; Zilioli et al., 2015), football players (Krenn and Meier, 2018) and so on.

However, with the development of the digital economy, the rise of e-sports has changed the landscape of the traditional sports industry and has also produced many e-sports players. As a new type of sport, e-sports are characterized by video gaming events in which professional and amateur players compete against each other. Its global market size grew dramatically from US$ 36.9 billion in 2015 to US$ 152.1 billion in 2019^1^.

Like traditional sports, e-sports players also need high-intensity fights to win, so they are also full of various aggressive behaviors in the game. Although there are some similarities between the two, the differences between e-sports and traditional sports also make the results of previous studies unable to be directly used in this research context. Unlike traditional sports, e-sports players do not directly engage in physical fighting, but use virtual characters as their representatives, which also occurs in the virtual world. Therefore, the two players do not need to meet directly, and they can only see the face of the other’s virtual characters. This change the research context of traditional sports that explain the relationship between person’s fWHR and their masculine behaviors that are related to dominant or aggressive.

Psychological research generally divides aggressive behavior into two categories based on motivation: hostile aggression and instrumental aggression. Hostile aggression is driven by emotions such as anger, with the primary intent to harm others. Instrumental aggression is deliberate and goal-oriented, where harm is a means to an end, such as winning a game or obtaining resources (Bushman and Anderson, 2001). In e-sports, aggressive acts more closely resemble instrumental aggression. Players aim to win matches, climb rankings, or secure in-game rewards; actions such as “killing” opponents are strategic moves toward these goals rather than attempts to cause real-world harm.

However, the literature has not always clearly distinguished between these two forms. Studies linking fWHR to aggression have often examined hostile aggression, such as responses to provocation (Carré and McCormick, 2008). Others have focused on instrumental aggression, relating fWHR to success in competitive fighting or to goal-driven behaviors like the pursuit of dominance (Zilioli et al., 2015). Many studies overlook the underlying motivation, which may contribute to inconsistent findings. Some scholars believe that fWHR can predict people’s aggressive behavior (Hehman et al., 2013a; Wen and Zheng, 2020; Zilioli et al., 2015). However, other scholars have found that people’s fWHR is not directly related to their aggressive behavior, and their relationship is affected by many different factors (Hehman et al., 2013b; Kosinski, 2017; MacDonell et al., 2018). Overall, empirical work on instrumental aggression remains limited, and it is often conflated with hostile aggression. Given that aggression in e-sports is predominantly instrumental, examining its relationship with fWHR in this context is both necessary and timely.

In order to fulfill these gaps, we used two studies to extend the current understanding of the relationship between the fWHR (of persons’ actual and virtual faces) and their behavioral tendencies. By using the competition data of professional players in *League of Legend* (LoL), one of the most popular MOBA games in the world, we can further understand the relationship among players’ real face, virtual characters’ fWHR, and their aggressive behavior. This paper also applies automated facial recognition algorithm to calculate fWHR. Our conclusions are more robust by combining manual measurements with algorithm-based calculations. The innovation of our paper is that we creatively considered the influence of the similarity between the real and virtual faces on players’ behavior in the virtual world. The conclusions will enhance our understanding of fWHR, virtual identity, and aggressive behavior.

## 2 Theory and hypothesis

### 2.1 The relationship between actual-self and virtual-self

Some scholars have already studied the development and influence of virtual identification in games. Many studies have verified that the virtual-self and actual-self may have some interaction (Dunn and Guadagno, 2012), and many scholars have proved that the virtual-self and virtual identity will influence people’s behavior in actual life. For example, virtual characters will affect people’s healthy behaviors in real life (Rheu et al., 2019). And if the virtual role is a heroic avatar, the operator will show higher pro-social actions (Yoon and Vargas, 2014). Correspondingly, some scholars have explored the influence of actual-self on virtual behaviors. For example, personality factors will influence the form of virtual-self discrepancy (Dunn and Guadagno, 2012), and actual-self-presence will affect people’s behavior in the virtual world (Jin, 2012).

However, these studies only focus on the one-way impact of actual and virtual identities and behaviors, which are the influence of actual identity on the behavior in the virtual world, and the influence of virtual identity on the behavior in the real world. The existing research has not paid enough attention to the similarity between the virtual-self and the actual-self. Some scholars tried to study the influence of the similarity of virtual- and real-self on their characteristics, social identity, self-esteem, self-efficiency and so on (Gabarnet et al., 2022; Wang et al., 2014), but did not explore how this similarity would affect people’s behavior. This provides an opportunity for our study.

### 2.2 Player’s fWHR and their aggressive behavior in the virtual world

In the context of e-sports, we think that the direct impact of fWHR on the players’ behavior is weaker. According to previous studies, the influence of fWHR on people’s judgment of aggressive behavior mainly comes from their direct perception of facial cues (Mason et al., 2006). This always studied in the traditional sports, and players can see each other’s face. Scholars have several explains for this. Some scholars have suggested that fWHR is affected by the levels of testosterone in men (Lefevre et al., 2013), thus high fWHR means high levels of testosterone, which will lead to more aggressive behaviors.

Whether in the sports competition or the research conducted in various areas, it is necessary to make intuitive judgments and feelings based on the face (Hehman et al., 2015). However, e-sports players face the computer screen rather than the face of their opponents. The faces of the opponents also appeared in the game as virtual characters. This context changes the foundation of previous research, which emphasizes that human behavior is based on direct observation of the facial structure of the other person.

### 2.3 The similarity of player’s two faces and aggressive behavior

The influence of the similarity between the players’ real face and the virtual character’s facial features on their aggressive behavior is also deserved to study. Following the previous logic, we believe that the fWHR of an e-sports player would not directly affect their behavior, we still think that there are more complex relationships between them. Based on the analysis above, we proposed that when the fWHR of an e-sports player’s real face is closer to the fWHR of their virtual character’s face, they are more likely to show aggressive behavior. This mechanism can be explained through Social Identity Theory (Turner et al., 1979), which explains how people’s sense of self is shaped by their group memberships. In organizational research, when the members think their self-image is similar to the image of their organization, they tend to identify themselves with the organization (Bhattacharya and Sen, 2003; Dutton et al., 1994). They will think that the organization is more attractive to them (Coyle-Shapiro and Morrow, 2006), and will also be more active and perform better in their work.

Similarly, when players find that the virtual character in the game is highly similar to their own, they are more inclined to regard their actual self and their virtual character as the same individual, and more inclined to think from the perspective of the character (Cohen, 2001). At the same time, the players will establish a new identification, which combines the self-concept in the real world and the virtual concept in the virtual world. The players begin to merge their personal identity with their virtual identity. This heightened identification motivates the player to adopt behaviors that uphold the positive status of that identity. Based on the relevant research on the Proteus effect, we can deduce that the higher the similarity between players and virtual characters, the easier the new identification will be recognized by players (Yee et al., 2009), and this virtual identification will affect players’ behavior.

Players determine the virtual character’s behavior in the game and share the feedback through the character in the virtual world. When the similarity between the player and the character is high, the player will think they have more control over the character because they will better recognize this new identification. This identification will also help the player to perceive the achievements or perceptions of characters in the virtual world (Klimmt Christoph, 2009). Like traditional sports, the winning of e-sports is also based on competition. Although in a virtual world, players still need to perform some violent acts, such as killing, destroying, to enhance their powers or game skills to gain relative advantages. Therefore, when the similarity between players and characters is high, players tend to help characters achieve higher achievements in the virtual world (Kartsanis and Murzyn, 2016), which will inevitably be achieved by bringing more aggressive behaviors. In addition, when the similarity is high, players are more inclined to obtain the temporary higher self-described level of strength and courage in the real world, which will enhance their confidence and perceive heightened power to win the game (Bowman et al., 2012). This will also bring more aggressive behavior in the virtual world.

Based on this analysis, we hypothesized:

*H1*: The higher the similarity between players actual face and virtual face, the more aggressive they will behave in the virtual world.

## 3 Materials and methods

### 3.1 Data sources

LoL is one of the most popular e-sports in the world. In 2021, the monthly live population of the game exceeded 180 million. The LoL Global Finals held in 2021 attracted 73.86 million people to watch simultaneously, and the average number of viewers per minute reached 30.60 million. In 2020, LoL had 1,320 professional players and 65 tournaments. LoL can be considered as a typical representative of e-sports in terms of both the number of spectators and the size of players. Therefore, this paper uses the data from LoL for research.

This paper uses the data of lol professional players who participated in professional competitions from January 1st, 2017, to September 1st, 2022, for analysis. The data in this article is obtained mainly from Leaguepedia^2^, which is a wiki website covers tournaments, teams, players, and personalities in LoL.

During this period, there are 1,325 players participated in professional competitions. According to the competition rules of LoL, all players should take clear frontal photos of their faces before the game, which provides convenience for us to obtain data. We take 2022 as the base year to collect the face photo information. While collecting data, we found that not all the players provided clear photos. Some players’ photo is sideways, and it is impossible to obtain clear the photo by reversing or twisting the photo angle (for example, the sideways photos do not show both of the two ears, which makes it difficult for us to measure the width of the face). For these players, we tried our best to obtain their photos from previous years to calculate their fWHR because an individual’s fWHR does not change significantly over time (Jia et al., 2014). After deleting the samples that could not obtain clear photos, we got 954 valid samples, which count for 72% of the full sample. The chosen players are not significantly different from the others in professional age, win rate and average performance in the game. For these players, we have a total of 31,606 virtual character-player-matched data.

### 3.2 The fWHR of the e-sports players

We used the *dlib* code provided by Davis King to recognize, rotate and locate faces^3^, and used the source code provided by Ties de Kok on Github to calculate fWHR of faces in photos^4^. We used Python 3.5 to calculate fWHR. The operation principle of the codes is similar to manual calculation, but the judgment standard is more unified. The algorithm-derived fWHR variable is used in the main analysis of this paper.

To ensure the robustness of the research, we also used ImageJ to calculate the fWHR manually (Carré and McCormick, 2008). Two research assistants picked the points in the face photo and measure the distance between the points in ImageJ blindly. A third research assistant will measure the photo again if the difference between the first two fWHR measures is greater than 5% (Kamiya et al., 2019). The average number of two or three fWHR were calculated as the final number of players fWHR. The fWHR calculated manually is highly correlated with the algorithm-based calculated (*r* = 0.961, *p* < 0.001). The results used manually calculated fWHR as independent variable were consistent with those obtained using the algorithm-based measure. To quantitatively assess the reliability of the manual measurements, we calculated the intraclass correlation coefficient (ICC) using a two-way random-effects model with absolute agreement (Koo and Li, 2016). The ICC for player photographs was 0.989 (p < 0.001), indicating excellent inter-rater reliability.

### 3.3 The fWHR of virtual characters in the e-sports

In order to measure whether players will choose virtual characters similar to themselves, we need to calculate the fWHR of all virtual characters first. After preliminary processing of virtual character photos, we obtained the facial images of all characters. According to the differences in characters’ facial features, we divided them into three categories.

The photo of the first type of character is shown in Supplementary Figure S1. Such characters are like human beings in the virtual world, and their facial structures are the same as those of human beings. Therefore, we can use the above method to calculate the fWHR of players to calculate the fWHR of characters. There are 101 first-type characters, accounting for 63.4% of all characters.

The photo of the second type of character is shown in Supplementary Figure S2. Such characters are not human, but their facial feature is obvious and similar to human beings. We can easily locate their eyes and month. Therefore, for such characters, we used ImageJ to calculate the fWHR manually (Carré and McCormick, 2008). Two research assistants picked the points in the face photo and measured the distance between the points in ImageJ blindly. A third research assistant will measure the photo again if the difference between the first two fWHR measures is greater than 5% (Kamiya et al., 2019). The average number of two or three fWHR was calculated as the final number of virtual character’s fWHR. There are 57 s-type characters, accounting for 35.4% of all characters.

The photo of the third type of character is shown in Supplementary Figure S3. Such characters have obvious faces but lack points that can be located to calculate fWHR. However, although their faces are not human, we can also recognize their eyes and mouth. To ensure the accuracy of fWHR calculation for such characters, we invited five research assistants who had played the LoL before to locate the faces of these characters. The average fWHR calculated by them is used as the fWHR of the character. There are 2 third-type characters, accounting for 1.2% of all characters. As in the previous measurements, we calculated the intraclass correlation coefficient (ICC) (Koo and Li, 2016). The ICC for the full set of virtual characters was 0.994 (*p* < 0.001), indicating excellent inter-rater reliability.

In order to ensure the robustness of the research, we also use the subsamples of the virtual characters. First, we only consider using first-type characters to avoid the impact of the lack of comparability between the non-human and human characters. In the data analysis, we delete the second and third types of characters used by players, and only use the first type of characters for calculation. In total, we obtained 20,370 role usage data for 954 players. After using the first type of characters only to calculate the virtual face fWHR of players, all the hypotheses are still verified (see Supplementary Table S2).

We also removed the data of the third-type virtual characters from the sample, and only consider the first two types of characters when calculating players’ usage of virtual characters. This measurement avoids inaccurate calculation due to the inclusion of virtual characters that cannot obtain exact face structure points. We obtained 31,287 role usage data in total. The relevant results remained unchanged. These results also proved the robustness of our hypotheses (see Supplementary Table S3).

### 3.4 The similarity of the two “faces” of e-sports players

The similarity of the two “faces” of e-sports players is the new independent variable in study 2. The independent variable and control variables were the same as in study 1. We first calculated the average fWHR of virtual characters used by each player as their virtual face fWHR. Then, we used the fWHR of players calculated in study 1 minus the fWHR of their virtual face as the indicator to measure the actual and virtual faces similarity. When this indicator is smaller than 0, it means that the player’s fWHR is smaller than the average value of their virtual characters’ fWHR; When this indicator is bigger than 0, it means that the player’s fWHR is bigger than the average value of their virtual characters’ fWHR. The closer this indicator is to 0, the similar the player’s real fWHR is to their virtual fWHR. This variable varies from −1.06 to 1.09.

Before the formal analysis, we used the player’s fWHR as a predictive variable to regress the virtual character’s fWHR. When we included the same control variables as those in Tables 1, 2, we found that the player’s fWHR had no significant effect on the fWHR of the virtual character (*p* > 0.100, see Supplementary Table S4), which indicates that the player would not deliberately choose characters similar to their own face. This also excludes the influence of the player’s individual preference on the results.

**Table 1:** The OLS results of study 1.

Variable	Kill points	Kill points ratio	Average gold per minutes	Gold ratio
	Model 1	Model 2	Model 3	Model 4
Players fWHR	0.077	0.000	0.984	−0.000
	(1.143)	(0.082)	(0.360)	(−0.084)
Play away home	0.007	0.003	−0.628	0.001
	(0.176)	(1.072)	(−0.369)	(0.790)
Professional age	0.032**	0.000	−0.472	−0.000
	(3.576)	(0.097)	(−1.317)	(−0.733)
All-star player	0.237**	−0.001	5.658**	0.000
	(4.501)	(−0.175)	(2.665)	(0.143)
Top liner	1.362**	0.104**	105.451**	0.060**
	(23.054)	(28.212)	(44.213)	(49.604)
Jungle	1.274**	0.102**	63.921**	0.037**
	(22.322)	(28.461)	(27.706)	(31.318)
Mid liner	2.091**	0.160**	118.647**	0.068**
	(35.498)	(43.416)	(49.890)	(56.062)
Bot liner	2.308**	0.169**	131.866**	0.074**
	(37.847)	(44.101)	(53.394)	(59.136)
Constant	0.998**	0.091**	267.012**	0.152**
	(6.734)	(9.816)	(44.564)	(49.888)
*N*	954	953	951	951
Adjust *R*^2^	0.664	0.733	0.807	0.838

**Means *p* < 0.01; * means *p* < 0.05. All tests are two-tailed. *t*-value in parentheses.

**Table 2:** The OLS results of study 2.

Variable	Kill points		Kill points ratio		Average gold per minutes		Gold ratio	
	Model 5	Model 6	Model 7	Model 8	Model 9	Model 10	Model 11	Model 12
Similarity of two faces	0.192**	0.186**	0.010*	0.009*	6.811**	6.571*	0.003*	0.003*
	(2.998)	(2.913)	(2.464)	(2.357)	(2.629)	(2.543)	(2.575)	(2.496)
Squared Similarity of two faces		−0.385*		−0.031**		−15.917*		−0.007*
		(−2.541)		(−3.315)		(−2.597)		(−2.370)
Play away home	0.005	0.001	0.003	0.002	−0.749	−0.949	0.001	0.001
	(0.126)	(0.013)	(0.999)	(0.856)	(−0.441)	(−0.560)	(0.706)	(0.599)
Professional age	0.035**	0.035**	0.000	0.000	−0.317	−0.282	0.000	0.000
	(3.938)	(4.042)	(0.558)	(0.685)	(−0.888)	(−0.792)	(−0.222)	(−0.132)
All-star player	0.232**	0.226**	−0.001	−0.002	5.404*	5.121*	0.000	0.000
	(4.444)	(4.322)	(−0.308)	(−0.478)	(2.558)	(2.428)	(−0.007)	(−0.130)
Top liner	1.382**	1.389**	0.105**	0.106**	106.204**	106.470**	0.060**	0.061**
	(23.321)	(23.475)	(28.396)	(28.661)	(44.361)	(44.567)	(49.732)	(49.910)
Jungle	1.258**	1.256**	0.101**	0.101**	63.415**	63.340**	0.036**	0.036**
	(22.081)	(22.108)	(28.294)	(28.395)	(27.525)	(27.573)	(31.161)	(31.205)
Mid liner	2.076**	2.077**	0.159**	0.160**	118.155**	118.198**	0.067**	0.067**
	(35.313)	(35.432)	(43.298)	(43.548)	(49.776)	(49.945)	(55.978)	(56.131)
Bot liner	2.268**	2.277**	0.167**	0.167**	130.465**	130.821**	0.073**	0.074**
	(36.474)	(36.662)	(42.703)	(43.044)	(51.809)	(52.031)	(57.444)	(57.628)
Constant	1.156**	1.190**	0.092**	0.095**	268.946**	270.358**	0.151**	0.152**
	(23.623)	(23.514)	(29.906)	(29.865)	(135.956)	(132.161)	(150.800)	(146.369)
Adjust *R*2	0.667	0.669	0.735	0.737	0.808	0.809	0.840	0.840
Δ*F*	239.181**	6.455*	330.240**	10.989**	501.513**	6.745**	622.132**	5.617*
*N*	954	954	953	953	951	951	951	951

** Means *p* < 0.01; * means *p* < 0.05. All tests are two-tailed. *t*-value in parentheses.

Besides, we also exclude the influence of the strength of the virtual character on the choice decisions of players. As the e-sports competition is a team competition, players may choose characters according to the team’s requirements to win. There is a possibility that players will choose certain characters based on the features of their team, which will lead to data deviation. Therefore, we use the fWHR of characters to conduct regression analysis on the characters’ victory rate. After controlling the category of the virtual role, the results showed no significant relationship between the fWHR of the virtual characters and their win rate (*N* = 161, *p* > 0.100, see Supplementary Table S5).

### 3.5 Players’ aggressive behavior

We use the relevant data from the competition to measure the players’ behavior. Different from traditional sports, the selection of the winner of e-sports like LoL does not rely on scores. Therefore, the scores of players are more like the indicators of their behavioral tendencies. Although this behavior is conducive to improving team performance, it is not the decisive factor in winning the game. For example, in a basketball game, the sum of players’ scores is the team’s score, and the team with higher scores will win. In LoL, players can also get a kill point after defeating an enemy. However, these points do not determine the team’s victory. The team’s victory or defeat depends on which team can destroy the opponent’s base first.

This rule allows us to infer the aggressive tendency of players according to their behaviors. In this study, aggression refers to instrumental aggression—goal-directed competitive behaviors such as kill points and gold acquisition—rather than hostile or emotional aggression (Bushman and Anderson, 2001). This perfectly describes many actions in a competitive e-sports economy. To exclude the influence of the competition and the team’s overall ability on the player’s behavior, we calculated several independent variables to measure the player’s aggressive behavior. First, we counted how many opponents each player killed in each game and calculated the average value of the player’s entire career as their *kill points*. If a player achieves more kill points in the game, he can be considered as more aggressive. In order to exclude the influence of team overall ability and performance, we also used the players’ *kill points ratio*, calculated by dividing the player’s kill points by the whole team’s kill points.

Second, we counted the gold obtained by the players in each game. In LoL rules, the more gold the player has, the more they can enhance their virtual character’s ability by purchasing props and be able to show more aggressive behavior in the game. Therefore, we also use these kinds of variables to measure the behavior tendency of players from another perspective.

Since the duration of LoL games are not fixed, and the gold is positively correlated to game time, we divide gold by the game time to obtain the gold per minute of players in each game, and calculate the average value of all games in their career. The larger the *average gold per minutes*, the greater the player’s ability to obtain resources. In competitive games, it is necessary to take greater risks to get more resources. Therefore, the larger this variable is, the more likely the players will be aggressive. Similarly, in order to control team’s ability, we calculated players *gold ratio* by dividing the player’s gold by the team’s gold.

### 3.6 Control variables

Although there is little research study the influence of fWHR of e-sports players on their aggressive behavior, there are a lot of studies explored the factors that potentially influence the relationship between fWHR and aggressive behavior in traditional sports. We introduced some control variables based on previous research.

The aggressive behavior of players will be affected by cultural factors. Players may be more likely to show their original behavior tendency in the familiar cultural environment. In contrast, in other cultural environments, players may be subject to various pressures to avoid aggressive behaviors (Koolhaas et al., 2007; Wisse and Sleebos, 2015). Therefore, we use a dummy variable to control whether the player is in a foreign team. This variable equals to 1 if players *play away home*.

The experience of players will also affect their competition behavior (Pifer et al., 2018). For active players, we calculated their *professional age* based on 2022. For the retired players, we calculated their *professional age* based on their actual years of joining and retiring. The bigger the *professional age*, the more experienced the players are.

Based on the previous studies, we controlled players’ *social status* (Krenn and Meier, 2018) using the dummy variable of whether the player had joined the all-star game. This variable equals to 1 if a player had joined the all-star game. For those players with higher social status, they are more willing to play aggressively to gain audience attention and attract more fans.

Several studies also found that players’ *positions* would influence their behavior. For example, fWHR will have a larger influence on player in attacking positions than in defense positions (Fujii and Takagishi, 2016; Welker et al., 2015). Thus, based on the positions of lol games, we created 4 dummy variables to control players’ positions, which are *top liner*, *jungle*, *mid liner*, and *bot liner*.

## 4 Results

### 4.1 Pretest results

We used the correlation and OLS regression to seek the relationship between e-sports players’ fWHR and their aggressive behavior. The descriptive and correlation matrix can be seen in Supplementary Table S1.

Table 3 showed the correlations between players’ fWHR and their aggressive behavior. The significance of all of the correlations is bigger than 0.05, and the confidence interval (CI) contains 0 at 95% level, which cannot prove a significant relationship. Sports players’ behavior will be influenced by their positions (Fujii and Takagishi, 2016). Thus, we also test the correlation of fWHR and aggressive behaviors for players in subsamples of different positions. As shown in Table 3, the significance of all the correlations is bigger than 0.05, which means there is no significant relationship between players’ fWHR and their aggressive behavior in subsamples.

**Table 3:** Correlations of fWHR and players aggressive behaviors.

Variable	*N*	Kill points			Kill points ratio			Average gold per minutes			Gold ratio		
		*r*	95% CI	*p*	*r*	95% CI	*p*	*r*	95% CI	*p*	*r*	95% CI	*p*
Full Sample	954	−0.001	[−0.065, 0.062]	0.974	−0.018	[−0.081, 0.046]	0.584	−0.004	[−0.067, 0.060]	0.904	−0.015	[−0.079, 0.049]	0.643
Subsamples of different positions:													
Top liner	186	0.032	[−0.112, 0.175]	0.665	0.122	[−0.022, 0.262]	0.096	0.130	[−0.014, 0.269]	0.076	0.138	[−0.006, 0.276]	0.061
Jungle	216	−0.060	[−0.192, 0.074]	0.377	−0.055	[−0.187, 0.079]	0.419	0.063	[−0.071, 0.195]	0.357	−0.008	[−0.142, 0.126]	0.904
Mid liner	189	0.132	[−0.011, 0.270]	0.070	−0.023	[−0.165, 0.120]	0.751	0.060	[−0.083, 0.201]	0.412	−0.068	[−0.209, 0.075]	0.350
Bot liner	165	0.040	[−0.113, 0.192]	0.606	0.046	[−0.108, 0.197]	0.563	−0.047	[−0.200, 0.107]	0.546	−0.002	[−0.156, 0.152]	0.978
Support	198	−0.016	[−0.155, 0.124]	0.821	−0.022	[−0.161, 0.118]	0.763	−0.013	[−0.152, 0.127]	0.860	0.002	[−0.138, 0.141]	0.979

CI = confidence interval, actual *p* reported.

Unlike traditional sports, e-sports involve no direct physical confrontation, which may limit the extent to which fWHR predicts aggression. This indicates that the effect of fWHR is weaker in competitive virtual settings.

The results of regressions are shown in Table 1. There is a slight variation in the number of samples between models, mainly because the dependent variables for some samples are not available. The effect of players’ fWHR did not turn out significant in all of the models (*p* > 0.100 for all the models). It is hard for us to prove that there is a significant relationship between e-sports players’ fWHR and their aggressive behavior. These results are consistent with many studies using the sample of traditional sports players (Kosinski, 2017).

### 4.2 Main results

We used the OLS method to test the hypothesis 1 in Table 2. Model 5, 7, 9 and 11 only contained the independent and control variables; Model 6, 8, 10 and 12 added the squared independent variable. The results show that if players tend to choose virtual characters with smaller fWHR than their real faces, they more tend to perform aggressive behaviors (Model 5: *β* = 0.192, 95% CI: [0.066, 0.318], *p* = 0.003; Model 7: *β* = 0.010, 95% CI: [0.002,0.018], *p* = 0.014; Model 9: *β* = 6.811, 95% CI: [1.727, 11.895], *p* = 0.009; Model 11: *β* = 0.003, 95% CI: [0.001, 0.006], *p* = 0.010). After adding the square of the similarity between the player’s actual and virtual faces’ fWHR, the coefficient of the squared variable is significantly negative (Model 6: *β* = −0.385, 95% CI: [−0.683, −0.088], *p* = 0.011; Model 8: *β* = −0.031, 95% CI: [−0.050, −0.013], *p* = 0.001; Model 10: *β* = −15.917, 95% CI: [−27.945, −3.890], *p* = 0.010; Model 12: *β* = −0.007, 95% CI: [−0.013, −0.001], *p* = 0.018). The results showed that when the difference between the fWHR of the player’s virtual character and their real face is small, the player will perform more aggressively. When the difference between the fWHR of the player’s virtual character and their real face is big, the player will show less aggressive behavior. These results verify H1.

**Figure 1:**
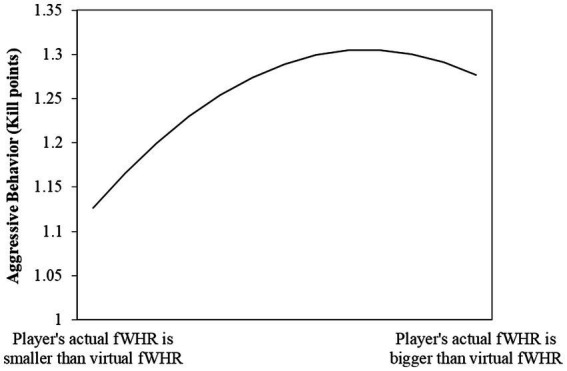
The inverse U-shape effect of the squared similarity of two faces of e-sports play.

Taking the results of Model 10 as an example, we draw the nonlinear relationship between the independent variable and the dependent variable to indicate effect sizes. As shown in Figure 1, there is an inverse U-shape relationship between the variables. We also use the U-test in stata to ensure the nonlinear effect is significant. The results show that when we use *kill points* as the dependent variable, the DV reaches the extreme point when IV = 0.242, and the presence of the inverse U-shape is significant (*p* < 0.05). This indicates that when the player’s real fWHR is slightly larger than the fWHR of their virtual character (around 10% larger), they will show the most aggressive behavior. The results were similar when we used the other three dependent variables.

In order to ensure the robustness of the results, we also calculated two variables to replace the independent variable. First, we calculated the ratio of the player’s actual fWHR to the average value of their virtual character’s fWHR. The closer the ratio is to 1, the closer the player’s fWHR is to their virtual fWHR. The use of ratio as the independent variable can avoid negative values and is more conducive to interpreting the practical meaning of the results. The results prove the robustness of the results (see Supplementary Table S6). Second, based on the fact that some players will prefer to choose certain characters, it is not accurate to calculate players’ virtual fWHR using simple average method. Therefore, we computed players’ virtual fWHR according to the frequency weighting of the virtual role used by the player. The results also remained unchanged (see Supplementary Table S7).

## 5 General discussion

The purpose of this study is to analyze the impact of fWHR on the aggressive behavior of e-sports players. We conducted the empirical study using the samples from LoL e-sports. The research conclusions of this paper have more reliable contributions based on the research context of MOBA e-sports represented by LoL. The competition rules of LoL enhance the possibility of separating team performance and individual behavior, which provides a possibility to further understand the relationship between the players’ fWHR and their behavior in team sports. In the traditional sports of scoring system (such as basketball or football), it is difficult for scholars to separate the individual behavior and team behavior of players. For example, if a football player scored, it could be considered both as their personal behavior tendency to perform aggressively, and also as the strategic requirement of the team. Therefore, it is difficult to define the individual behavior of players in traditional sports accurately. However, e-sports like LoL separate the player’s and team’s performance through rules-making. Team performance is measured by the destruction of opponents’ base, which is correlated with, but not determined by player’s behavior. Players’ killing and other behaviors are more like the reflection of their behavioral tendencies. This research context can help us better understand the relationship between the player’s fWHR and their behavior.

This article has two main findings: First, we did not find any impact of fWHR on e-sports players’ aggressive behavior. There are certain connections and differences between e-sports and traditional sports. Like traditional competitive sports, e-sports also require players to conduct high-intensity confrontations. In the professional competition of e-sports, male players also occupy a dominant position. Scholars have found that fWHR was affected by the testosterone levels in men (Lefevre et al., 2013), thus, men with higher fWHR may have more aggressive behavior.

However, some scholars have also proved that fWHR cannot predict aggressive behaviors directly, whether for traditional sports athletes or people in other fields (Kosinski, 2017; Krenn and Meier, 2018; Stefanidis et al., 2022; Wang et al., 2019). The special features of e-sports further weaken the relationship between player’s fWHR and their aggressive behavior. E-sports is not a direct physical confrontation between players. The competition requires players to operate virtual characters on the game platform. This weakens the direct impact of fWHR on players’ aggressive behavior. The results of the empirical analysis also show that there is no significant relationship between fWHR and players’ aggressive behavior.

In addition, in order to get the robust results, we controlled players’ positions, social status, social pressure and experience according to previous studies. E-sports also has some unique advantages in data obtaining. For example, the detailed data of players, teams and games will be recorded in digital form, which is more accessible than in traditional sports. In this paper, we only used control variables based on the suggestions of previous studies. These variables are enough to help us explain the research question of our paper. However, e-sports can provide more dimensional detailed information. In future studies, more variables should be used to expand the research further.

Second, we found that when e-sports players use virtual characters that are more similar to their actual facial structures, they tend to show higher aggressive behaviors. This conclusion enhances our understanding of virtual identity in the digital economy era. With the development of the internet, virtual identity has become an essential part of people’s life. Many actions can be conducted without a real identity. Therefore, many behaviors that require information based on people’s real face are now impossible. However, this study found that even if people use virtual faces in the virtual world, real faces will also impact people’s behavior through the construction of self-cognition and new identities. Based on the results using the samples of e-sports players, we found that when people’s virtual face and real face are highly similar, they tend to behave more aggressively in the virtual world. Our studies contribute to the research of virtual identity. Many scholars have verified the impact of the virtual self on their actual behavior, but few pay attention to people’s behavior in the virtual world. As the virtual world becomes increasingly important, we should pay more attention to the impact of actual and virtual self-identity on virtual world behavior in further research.

Moreover, our analyses revealed an inverse U-shaped relationship between player–virtual character fWHR similarity and instrumental aggression. A plausible explanation lies in the Proteus Effect (Yee and Bailenson, 2007), which holds that individuals’ behaviors align with their digital self-representations. Through this process, people infer an identity from their virtual characters and act in ways consistent with that identity. In our context, players may partially internalize the dominance-related cues associated with a higher fWHR, thereby shaping their in-game behavior. The Proteus Effect can be viewed as a specific application of Self-Perception Theory in virtual settings (Coesel et al., 2024). When similarity between player and avatar is very low, there is insufficient alignment to trigger self-perception processes. The peak aggression observed may reflect optimal identification (Brewer, 1991), where the avatar is seen as both self-resembling and slightly idealized, creating an “enhanced self” that boosts confidence and competitive drive. Conversely, when similarity is extremely high and the avatar is virtually identical to the player, the need for uniqueness is not met (Brewer, 1991; Leonardelli et al., 2010), and aggression is less likely to be expressed in line with the virtual identity. Future research could further examine these boundary conditions in competitive virtual environments.

Besides, although we did not report some results in detail due to the page limit, we have used empirical studies to prove that e-sports players do not deliberately choose virtual characters which are more similar to their own. This shows that for most players, they do not realize that the character selection will affect their own behavior. But after character selection, players will decide whether to conduct more aggressive behavior according to the similarity of the virtual face. Therefore, we need to further explore whether people’s subjective intentions of virtual faces choice will impact their virtual behavior. We also conducted some studies to avoid the effects of players’ preference of virtual characters on the results. We found that even if players chose a virtual character more like themselves, the winning rate of the players did not improve significantly. The results showed that the similarity between virtual faces and real faces did not improve team performance, but only affected players’ behavior. In the future research, we should further analyze these phenomena and build a complete theoretical framework to understand these relationships better.

In conclusion, in the context of e-sports, we have verified that the players’ facial structure has no direct connection with their behavior. But when the similarity between players and their virtual characters is higher, they will show more aggressive behavior. These conclusions enhance our understanding of the relationship among appearance, identity, and behavior. While our results and conclusions appear robust, we also want to acknowledge two limitations: First, because of the particularity of virtual characters, although we used a variety of robustness tests, we were still not certain whether the measurement of the non-human virtual characters’ face structure is reliable. We have tried our best to avoid the influence of the measurement on our results, but this still needs further development. Second, although LoL is one of the most influential and representative e-sports in the world, there are some differences between different types of e-sports, just like the differences between different types of traditional sports. Whether the relevant conclusions can be extended to other e-sports is still worth further exploration. We think future research can extend our work to consider these and other issues, and maybe a new theory about virtual face, identity and behavior can be developed to better explain our studies.

## Data Availability

The raw data supporting the conclusions of this article will be made available by the authors, without undue reservation.

## Footnotes

^1^Data from https://newzoo.com/insights/trend-reports/newzoo-global-games-market-report-2019-light-version/.

^2^Leaguepedia | League of Legends Esports Wiki (https://fandom.com/), accessible at https://lol.fandom.com/wiki/League_of_Legends_Esports_Wiki.

^3^GitHub–ageitgey/face_recognition: The world’s simplest facial recognition api for Python and the command line, accessible at https://github.com/ageitgey/face_recognition.

^4^GitHub–CarlLindersson/fWHR_Calculator: Program to collect sports athletes’ facial width-to-height ratio (fWHR) and statistics by web scraping, accessible at https://github.com/CarlLindersson/fWHR_Calculator.
