# Supplementary material for: Two “faces” of e-sports players: the relationship between facial width-to-height ratio and aggressive behavior in the virtual world
Source: Front Psychol. 2025 Nov 19;16:1620182. doi: 10.3389/fpsyg.2025.1620182 (PMC12672536; doi:10.3389/fpsyg.2025.1620182)
Supplement: Supplementary file 1 [file Table_1.DOCX]

**Supplemental Materials**

**Table S1.** Descriptive and Correlation matrix

|  | Mean | S.D. | 1 | 2 | 3 | 4 | 5 | 6 | 7 | 8 |
| --- | --- | --- | --- | --- | --- | --- | --- | --- | --- | --- |
| 1. Kill Points | 2.609 | 0.997 |  |  |  |  |  |  |  |  |
| 2. Players fWHR | 1.984 | 0.284 | -0.001 |  |  |  |  |  |  |  |
| 3. Play away home | 0.273 | 0.445 | 0.037 | 0.039 |  |  |  |  |  |  |
| 4. Professional Age | 2.788 | 2.176 | 0.090** | -0.196** | 0.002 |  |  |  |  |  |
| 5. All-Star Player | 0.000 | 0.358 | 0.088** | 0.072** | 0.058* | 0.007 |  |  |  |  |
| 6. Top liner | 0.195 | 0.396 | -0.005 | 0.003 | -0.028 | -0.037 | 0.007 |  |  |  |
| 7. Jungle | 0.226 | 0.419 | -0.058* | -0.015 | 0.029 | -0.082** | -0.018 | -0.266** |  |  |
| 8. Mid liner | 0.198 | 0.399 | 0.367** | -0.042 | 0.027 | 0.054* | 0.040 | -0.245** | -0.269** |  |
| 9. Bot liner | 0.173 | 0.378 | 0.433** | 0.030 | 0.006 | 0.050 | -0.030 | -0.225** | -0.247** | -0.227** |

Notes: **means p<0.01; *means p<0.05. Two-tailed test.

**Table S2.** OLS results of first category of virtual characters only

|  | Kill points | | Kill points ratio | | Average gold per minutes | | Gold ratio | |
| --- | --- | --- | --- | --- | --- | --- | --- | --- |
|  | Model 9 | Model 10 | Model 11 | Model 12 | Model 13 | Model 14 | Model 15 | Model 16 |
| Similarity of | 0.173* | 0.287** | 0.008 | 0.015** | 6.404* | 10.160** | 0.003* | 0.005** |
| two faces | (2.437) | (3.547) | (1.661) | (2.597) | (2.273) | (3.149) | (2.380) | (3.038) |
| Squared Similarity |  | -0.512** |  | -0.030* |  | -16.643* |  | -0.007* |
| of two faces |  | (-2.905) |  | (-2.356) |  | (-2.370) |  | (-1.958) |
| Play away home | 0.021 | 0.016 | 0.004 | 0.004 | -0.561 | -0.721 | 0.001 | 0.000 |
|  | (0.467) | (0.357) | (1.213) | (1.123) | (-0.313) | (-0.403) | (0.591) | (0.517) |
| Professional Age | 0.034** | 0.035** | -0.000 | -0.000 | -0.239 | -0.214 | -0.000 | 0.000 |
|  | (3.585) | (3.681) | (-0.514) | (-0.442) | (-0.634) | (-0.568) | (-0.030) | (0.026) |
| All-Star Player | 0.239** | 0.232** | -0.002 | -0.002 | 5.672* | 5.460 | -0.000 | -0.000 |
|  | (4.234) | (4.131) | (-0.392) | (-0.488) | (2.544) | (2.454) | (-0.188) | (-0.267) |
| Top liner | 1.442** | 1.446** | -0.099** | -0.099** | 111.088** | 111.199 | 0.062** | 0.062** |
|  | (22.618) | (22.771) | (-21.797) | (-21.785) | (44.107) | (44.252) | (49.246) | (49.348) |
| Jungle | 1.386** | 1.391** | 0.000 | -0.000 | 65.354** | 65.518 | 0.038** | 0.038** |
|  | (22.486) | (22.644) | (-0.077) | (-0.013) | (26.834) | (26.957) | (30.792) | (30.882) |
| Mid liner | 2.095** | 2.101** | -0.024** | -0.024** | 118.757** | 118.967 | 0.067** | 0.067** |
|  | (33.057) | (33.270) | (-5.374) | (-5.299) | (47.485) | (47.656) | (53.450) | (53.567) |
| Bot liner | 2.289** | 2.295** | -0.020** | -0.020** | 131.852** | 132.049 | 0.074** | 0.074** |
|  | (34.636) | (34.845) | (-4.278) | (-4.211) | (50.330) | (50.503) | (56.348) | (56.467) |
| Constant | 1.080** | 1.116** | 0.684** | 0.686** | 268.212** | 269.362** | 0.152** | 0.152** |
|  | (19.750) | (19.982) | (175.354) | (172.058) | (124.059) | (121.848) | (139.939) | (137.158) |
| Adjust R2 | 0.633 | 0.666 | 0.407 | 0.410 | 0.796 | 0.797 | 0.830 | 0.830 |
| ΔF | 206.383** | 8.439** | 82.758** | 5.551* | 464.009** | 5.618* | 578.706** | 3.835* |
| N | 954 | 954 | 953 | 953 | 951 | 951 | 951 | 951 |

Notes: **means *p*<0.01; *means *p*<0.05. All tests are two-tailed. t-value in parentheses.

**Table S3.** OLS results of first and second category of virtual characters

|  | Kill points | | Kill points ratio | | Average gold per minutes | | Gold ratio | |
| --- | --- | --- | --- | --- | --- | --- | --- | --- |
|  | Model | Model | Model | Model | Model | Model | Model | Model |
| Similarity of | 0.192** | 0.180** | 0.010* | 0.009* | 6.957** | 6.468* | 0.003** | 0.003* |
| two faces | (2.986) | (2.798) | (2.559) | (2.328) | (2.676) | (2.487) | (2.628) | (2.461) |
| Squared Similarity |  | -0.376** |  | -0.030** |  | -15.242* |  | -0.007* |
| of two faces |  | (-2.445) |  | (-3.071) |  | (-2.455) |  | (-2.149) |
| Play away home | 0.008 | 0.003 | 0.003 | 0.002 | -0.770 | -0.962 | 0.001 | 0.001 |
|  | (0.184) | (0.073) | (1.075) | (0.940) | (-0.453) | (-0.567) | (0.685) | (0.586) |
| Professional Age | 0.035** | 0.035** | 0.000 | 0.000 | -0.315 | -0.286 | -0.000 | -0.000 |
|  | (3.936) | (4.026) | (0.610) | (0.717) | (-0.883) | (-0.801) | (-0.210) | (-0.137) |
| All-Star Player | 0.233** | 0.227** | -0.001 | -0.001 | 5.306* | 5.054* | 0.000 | -0.000 |
|  | (4.443) | (4.332) | (-0.228) | (-0.377) | (2.507) | (2.392) | (0.005) | (-0.099) |
| Top liner | 1.386** | 1.392** | 0.106** | 0.106** | 106.292** | 106.518** | 0.061** | 0.061** |
|  | (23.355) | (23.493) | (28.475) | (28.699) | (44.329) | (44.509) | (49.712) | (49.853) |
| Jungle | 1.262** | 1.260** | 0.101** | 0.101** | 63.690** | 63.608** | 0.037** | 0.037** |
|  | (22.161) | (22.179) | (28.392) | (28.467) | (27.664) | (27.699) | (31.310) | (31.336) |
| Mid liner | 2.072** | 2.073** | 0.159** | 0.160** | 118.133** | 118.192** | 0.067** | 0.068** |
|  | (35.186) | (35.301) | (43.201) | (43.422) | (49.666) | (49.821) | (55.895) | (56.021) |
| Bot liner | 2.272** | 2.281** | 0.167** | 0.168** | 130.469** | 130.857** | 0.073** | 0.074** |
|  | (36.454) | (36.631) | (42.668) | (42.966) | (51.697) | (51.887) | (57.355) | (57.487) |
| Constant | 1.118** | 1.152** | 0.092** | 0.094** | 268.134** | 269.520** | 0.151** | 0.152** |
|  | (22.583) | (22.453) | (29.472) | (29.324) | (133.945) | (129.908) | (149.033) | (144.275) |
| Adjust R2 | 0.666 | 0.668 | 0.734 | 0.737 | 0.808 | 0.809 | 0.839 | 0.840 |
| ΔF | 238.784** | 5.980* | 330.131** | 9.434** | 500.289** | 6.029* | 621.389** | 4.620* |
| N | 954 | 954 | 953 | 953 | 951 | 951 | 951 | 951 |

Notes: **means *p*<0.01; *means *p*<0.05. All tests are two-tailed. t-value in parentheses.

**Table S4.** Regression results of players’ fWHR on their virtual characters’ fWHR

|  | The fWHR of Virtual Characters |
| --- | --- |
|  | Model |
| Players fWHR | -0.013 |
|  | (-1.462) |
| Play away home | 0.003 |
|  | (0.567) |
| Professional Age | 0.000 |
|  | (-0.207) |
| All-Star Player | 0.013 |
|  | (1.845) |
| Top liner | 0.093** |
|  | (11.718) |
| Jungle | -0.104** |
|  | (-13.511) |
| Mid liner | -0.105** |
|  | (-13.220) |
| Bot liner | -0.201** |
|  | (-24.431) |
| Constant | 2.081** |
|  | (104.115) |
| N | 954 |
| Adjust R2 | 0.613 |

Notes: **means *p*<0.01; *means *p*<0.05. All tests are two-tailed. t-value in parentheses.

**Table S5.** Regression results of virtual characters’ fWHR on their win rate

|  | The win rate of Virtual Characteristics |
| --- | --- |
|  | Model |
| Virtual Characters’ fWHR | -0.005 |
|  | (-0.789) |
| Fighter | -0.020 |
|  | (-2.126) |
| Tank | -0.002* |
|  | (-0.189) |
| Mage | -0.020 |
|  | (-1.919) |
| Assassin | -0.007 |
|  | (-0.713) |
| Marksman | -0.006 |
|  | (-0.534) |
| Constant | 0.518** |
|  | (31.445) |
| N | 161 |
| Adjust R2 | 0.014 |

Notes: **means *p*<0.01; *means *p*<0.05. All tests are two-tailed. t-value in parentheses.

**Table S6.** OLS results using the ratio of the player's actual fWHR to the average value of his virtual character's fWHR as independent variable

|  | Kill points | | Kill points ratio | | Average gold per minutes | | Gold ratio | |
| --- | --- | --- | --- | --- | --- | --- | --- | --- |
|  | Model | Model | Model | Model | Model | Model | Model | Model |
| Similarity of | 0.367** | 3.914** | 0.018* | 0.251** | 12.887* | 139.866** | 0.007* | 0.065* |
| two faces (ratio) | (2.872) | (3.149) | (2.263) | (3.221) | (2.495) | (2.782) | (2.508) | (2.550) |
| Squared Similarity |  | -1.752** |  | -0.115** |  | -62.712* |  | -0.029* |
| of two faces (ratio) |  | (-2.869) |  | (-3.004) |  | (-2.539) |  | (-2.305) |
| Play away home | 0.006 | -0.001 | 0.003 | 0.002 | -0.732 | -0.963 | 0.001 | 0.001 |
|  | (0.136) | (-0.014) | (1.010) | (0.855) | (-0.431) | (-0.568) | (0.714) | (0.591) |
| Professional Age | 0.034** | 0.035** | 0.000 | 0.000 | -0.326 | -0.294 | -0.000 | -0.000 |
|  | (3.913) | (4.020) | (0.519) | (0.625) | (-0.913) | (-0.826) | (-0.234) | (-0.154) |
| All-Star Player | 0.233** | 0.225** | -0.001 | -0.002 | 5.442** | 5.121* | 0.000 | -0.000 |
|  | (4.462) | (4.302) | (-0.287) | (-0.466) | (2.576) | (2.427) | (0.007) | (-0.131) |
| Top liner | 1.380** | 1.384** | 0.105** | 0.106** | 106.095** | 106.242** | 0.060** | 0.060** |
|  | (23.295) | (23.446) | (28.370) | (28.554) | (44.361) | (44.537) | (49.754) | (49.909) |
| Jungle | 1.259** | 1.259** | 0.101** | 0.101** | 63.454** | 63.440** | 0.036** | 0.036** |
|  | (22.095) | (22.169) | (28.307) | (28.417) | (27.537) | (27.610) | (31.174) | (31.239) |
| Mid liner | 2.077** | 2.081** | 0.160** | 0.160** | 118.199** | 118.369** | 0.067** | 0.068** |
|  | (35.327) | (35.529) | (43.309) | (43.560) | (49.789) | (49.984) | (55.996) | (56.166) |
| Bot liner | 2.268** | 2.284** | 0.167** | 0.168** | 130.470** | 131.066** | 0.073** | 0.074** |
|  | (36.370) | (36.619) | (42.602) | (42.878) | (51.673) | (51.834) | (57.289) | (57.386) |
| Constant | 0.788** | -0.968 | 0.074** | -0.042 | 256.026** | 193.146** | 0.145** | 0.116** |
|  | (5.708) | (-1.544) | (8.497) | (-1.059) | (45.863) | (7.609) | (51.120) | (8.988) |
| Adjust R2 | 0.666 | 0.669 | 0.734 | 0.739 | 0.808 | 0.809 | 0.839 | 0.840 |
| ΔF | 238.905** | 8.232** | 329.791** | 9.025** | 501.063** | 6.447* | 621.865** | 5.312* |
| N | 954 | 954 | 953 | 953 | 951 | 951 | 951 | 951 |

Notes: **means *p*<0.01; *means *p*<0.05. All tests are two-tailed. t-value in parentheses.

**Table S7.** OLS results using the players’ virtual fWHR according to the frequency weighting of the virtual role used by the player to calculate the independent variable

|  | Kill points | | Kill points ratio | | Average gold per minutes | | Gold ratio | |
| --- | --- | --- | --- | --- | --- | --- | --- | --- |
|  | Model | Model | Model | Model | Model | Model | Model | Model |
| Similarity of | 0.168** | 0.174** | 0.009* | 0.009* | 5.918* | 6.177* | 0.003* | 0.003* |
| two faces (weighted) | (2.662) | (2.757) | (2.173) | (2.350) | (2.315) | (2.418) | (2.205) | (2.318) |
| Squared Similarity |  | -0.259† |  | -0.028** |  | -11.111* |  | -0.006* |
| of two faces (weighted) |  | (-1.951) |  | (-3.430) |  | (-2.069) |  | (-2.267) |
| Play away home | 0.005 | 0.002 | 0.003 | 0.002 | -0.744 | -0.893 | 0.001 | 0.001 |
|  | (0.129) | (0.048) | (1.002) | (0.864) | (-0.438) | (-0.526) | (0.711) | (0.615) |
| Professional Age | 0.034** | 0.034** | 0.000 | 0.000 | -0.352 | -0.328 | -0.000 | -0.000 |
|  | (3.842) | (3.905) | (0.468) | (0.579) | (-0.987) | (-0.923) | (-0.329) | (-0.257) |
| All-Star Player | 0.233** | 0.229** | -0.001 | -0.001 | 5.414* | 5.257* | 0.000 | -0.000 |
|  | (4.443) | (4.378) | (-0.302) | (-0.426) | (2.560) | (2.489) | (0.001) | (-0.080) |
| Top liner | 1.404** | 1.419** | 0.107** | 0.108** | 106.969** | 107.612** | 0.061** | 0.061** |
|  | (22.970) | (23.068) | (27.830) | (28.198) | (43.327) | (43.321) | (48.507) | (48.515) |
| Jungle | 1.269** | 1.269** | 0.102** | 0.102** | 63.792** | 63.784** | 0.037** | 0.037** |
|  | (22.306) | (22.335) | (28.506) | (28.660) | (27.745) | (27.790) | (31.384) | (31.449) |
| Mid liner | 2.084** | 2.085** | 0.160** | 0.160** | 118.447** | 118.500** | 0.068** | 0.068** |
|  | (35.495) | (35.567) | (43.478) | (43.760) | (49.969) | (50.075) | (56.172) | (56.317) |
| Bot liner | 2.275** | 2.286** | 0.167** | 0.168** | 130.682** | 131.168** | 0.074** | 0.074** |
|  | (36.607) | (36.687) | (42.834) | (43.211) | (51.946) | (52.003) | (57.591) | (57.678) |
| Constant | 1.144** | 1.167** | 0.091** | 0.094** | 268.551** | 269.529** | 0.151** | 0.152** |
|  | (23.237) | (23.089) | (29.528) | (29.683) | (134.878) | (131.924) | (149.620) | (146.403) |
| Adjust R2 | 0.666 | 0.667 | 0.734 | 0.737 | 0.808 | 0.809 | 0.839 | 0.840 |
| ΔF | 238.469** | 3.805† | 329.602** | 11.767** | 500.501** | 4.283* | 620.750** | 5.139* |
| N | 954 | 954 | 953 | 953 | 951 | 951 | 951 | 951 |

Notes: **means *p*<0.01; *means *p*<0.05; †means *p*<0.10. All tests are two-tailed. t-value in parentheses.


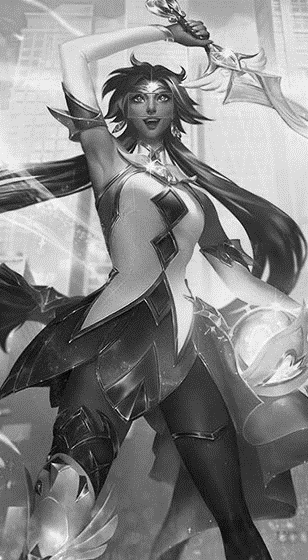


**Fig. S1.** Typical photo of the virtual character like human beings


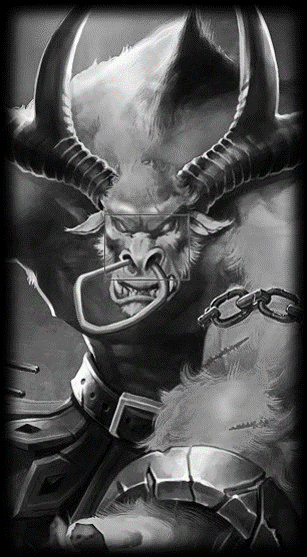


**Fig. S2.** Typical photo of the virtual character which is not a human but with obvious eyes, ears and mouth


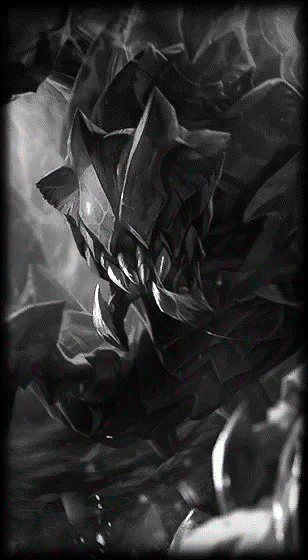


**Fig. S3.** Typical photo of the virtual character which is not a human and without obvious eyes, ears or mouth
